# Combination of conditional cash transfer program and environmental health interventions reduces child mortality: an ecological study of Brazilian municipalities

**DOI:** 10.1186/s12889-021-10649-4

**Published:** 2021-03-31

**Authors:** Anelise Andrade de Souza, Sueli Aparecida Mingoti, Rômulo Paes-Sousa, Leo Heller

**Affiliations:** 1 René Rachou Institute, Fiocruz Minas, Avenida Augusto de Lima, 1715, Barro Preto, Belo Horizonte, Minas Gerais Brazil; 2 Department of Statistics, Federal University of Minas Gerais, Belo Horizonte, Minas Gerais Brazil

**Keywords:** Water, Sanitation, Solid waste, Social programs, Interaction, Mortality

## Abstract

**Background:**

This study aims to assess the interactive effects of Brazilian public interventions, environmental health programs (access to water, sanitation and solid waste collection) and a Conditional Cash Transfer Program (PBF), on the mortality reduction due to diarrhea and malnutrition among children under 5 years old.

**Methods:**

The study design is ecological, with longitudinal analysis in a balanced panel. The period covered is 2006 to 2016, including 3467 municipalities from all regions of the country, which resulted in 38,137 observations. The generalized linear models were adjusted considering the Negative Binomial (NB) distribution for the number of deaths due to malnutrition and diarrhea, with fixed effects. NB models with and without zero-inflation were assessed. Subsequent interaction models were applied to assess the combined effects of the two public policies.

**Results:**

In relation to the decline of mortality rates due to diarrhea in the municipalities, positive effect modification were observed in the presence of: high coverage of the target population by the PBF and access to water, 0.54 (0.28–1.04) / 0.55 (0.29–1.04); high coverage by the total population by the PBF and access to water, 0.97 (0.95–1.00) and high coverage by the total population by the PBF and access to sanitation, 0.98 (0.97–1.00). Decline on diarrhea mortality was also observed in the joint presence of high coverage of solid waste collection and access to water, categories 1 (> 60% ≤85%): 0.98 (0.96–1.00), 0.98 (0.97–1, 00) and 2 (> 85% ≤ 100%): 0.97 (0.95–0.98), 0.97 (0.95–0.99). Negative effect modification were observed for mortality due to malnutrition in the presence of simultaneous high coverage of the total population by the PBF and access to sanitation categories 1 (≥ 20 < 50%): 1.0061 (0.9991–1.0132) and 2 (≥ 50 < 100%): 1.0073 (1.0002–1.0145) and high coverage of the total population by the PBF and solid waste collection, 1.0004 (1.0002–1.0005), resulting in malnutrition mortality rates increase.

**Conclusion:**

Implementation of environmental health services and the coverage expansion by the PBF may enhance the prevention of early deaths in children under 5 years old due to diarrhea, a poverty related disease.

**Supplementary Information:**

The online version contains supplementary material available at 10.1186/s12889-021-10649-4.

## Introduction

Brazil remains among the countries with the highest levels of inequality [1, 2]. Progress in poverty reduction achieved until 2014 began to reverse due to economic slowdown, after a period of sharp and sustained decline of poverty and inequality since 2004 [1, 2]. By the period when this study was carried out, the number of families living with limited access to basic goods and services was raising. The list of deficits encompasses income, food, adequate housing, and public services, such as health, education, water, sanitation and solid waste collection [2, 3].

In relation to environmental health interventions, the main deficits that still prevail in the country are primarily related to sanitation, which is far from the necessary level of universalization [3–6]. Houses connected to piped water in premises (from water systems or wells) responds boldly for 95.6% of Brazilian households [3]. However, when considering only those holding adequate access, according to “National Basic Sanitation Plan” (PLANSAB) [3], that coverage falls to 57.7% [3]. Only 48% of the households have suitable access to the sewerage system [3]. Finally, for solid waste collection, 64.9% of the population have access to urban cleaning and proper waste management [3]. The populations most affected by these inadequate conditions are those living in peri-urban and rural areas, i.e., the poorest population groups and, consequently, the most vulnerable [7]. Thus, the economic and social vulnerability of a large part of the Brazilian population makes these groups more likely to maintain the cycle of poverty-diseases. Therefore, transformative public policies need to be successfully implemented to break that pervasive chain of events [8–11],, requiring an integrated set of interventions to reduce vulnerabilities and risk factors associated with health inequities [8, 11].

In 2003, the Brazilian government created the Bolsa Família Program (PBF) aiming to reduce economic and social vulnerability. The PBF is a Conditional Cash Transfer Program (CCT) based on three dimensions: (i) cash transfer, with values that vary according to household income and family composition; (ii) conditionality, which work as incentives for beneficiaries to increase access to public health, social assistance and education services; (iii) complementary actions, which refer to other social programs offered to PBF beneficiaries [12]. In the short term, the main objective of this Program was poverty alleviation and improvement of food security [13]. The Program started in 2003 covering 3.6 million families, jumping to 11.2 million families in 2006. In 2019, all 5570 Brazilian municipalities had already implemented the PBF, benefiting 13.8 million families [14, 15].

Intersectoral social policies can result in gains for the population as a consequence of a better organization of the public offer. That is reflected in logistics improvement and better territorial focus [16].

In environmental health, the study of interactive effects with other interventions are important for assessing the combined effects of public interventions to increase the access to quality water, adequate sanitation and collection of solid waste. The extent to which improvements on health, education, social assistance and nutrition combined with interventions in water and sanitation still requires further development in a variety of contexts [17–22]. Thus, the objective of the current study is to assess the hypothesis that, the simultaneous presence of better access conditions to environmental health interventions (water, sanitation and solid waste collection) and the access to the PBF generates an interaction effect in reducing mortality due to malnutrition and diarrhea in children under 5 years old, which are responsible for significant number of deaths in Brazil, especially in the Northeast and North regions of the country [23–25].

## Methods

### Study design

This research is framed as an ecological design with exploratory and analytical analysis. The design allows for assessing the progress of the rates of an event in different population groups and to assess the association between the average level of exposure and the rates of an event between different population groups, over time [26].

In the current study, data from 3467 Brazilian municipalities were observed for each year in the 2006–2016 period. Municipalities, from different regions of the country, were followed in all 11 years according to their geographical delimitation in the beginning of the observation. New municipalities that emerged in the observed period were treated in the level of geographical aggregation presented in 2006. Thus, there is a delineation in balanced panel data, with the municipality being the unit of analysis resulting on 38,137 observations.

In this way, it was possible to perform a longitudinal study to assess the temporal changes of mortality rates due to malnutrition and diarrhea. Furthermore, association between the average exposure to independent variables (access to PBF and environmental health variables), and mortality rates of children under 5 years old was investigated. Finally, the design allowed for assess interaction between independent variables, mainly access to PBF and environmental health variables.

### Inclusion criteria for municipalities

In 2006, the starting year of the study, Brazil presented 5560 municipalities. All municipalities at the time presented: (i) adequacy of vital statistics data [27, 28]; (ii) annual data of mortality due to diarrhea and malnutrition for children under 5 years old; (iii) data on coverage of the target population and municipal total by the PBF; (iv) data on coverage by environmental health services (water, sanitation and solid waste collection) for the years 2000 and 2010.

To assess the adequacy of vital statistics data, a multidimensional criterion was applied [27, 28]. This criterion was used based on the following indicators, using their means and Confidence Intervals for the years 2006 to 2008: (i) Age-Standardized Mortality Rate (SMR); (ii) relative average deviation from SMR; (iii) ratio of informed and estimated live births; (iv) relative average deviation in birth rates; (v) proportion of deaths classified as ill-defined causes. Subsequently, according to the assumed criterion, the municipalities were classified according to data reliability as “satisfactory”, “unsatisfactory” and “deficient”. Only municipalities classified as “satisfactory” were selected for the study. The period used for assessing the data reliability was 2006 to 2008. The same classification of the municipalities was replicated for the years 2009 to 2016 [27, 28].

### Outcome variables

**Fig. 1:**
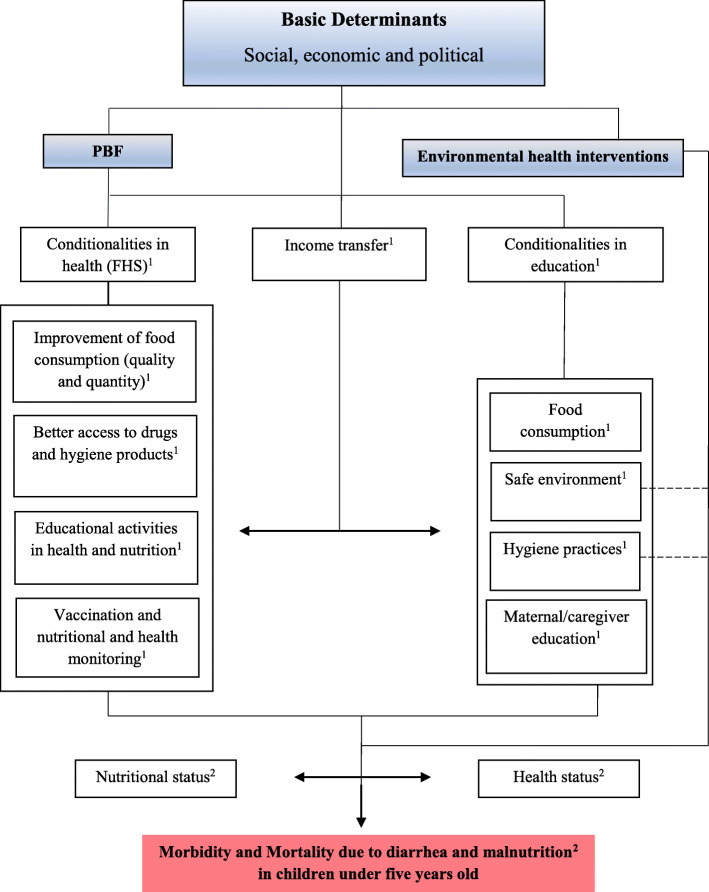
Mechanisms that link the PBF and access to environmental health interventions to children’s nutritional and health outcomes. Source: adapted from Groot et al. [29], and Rasella et al. [22],. ^1^Underlying determinants. ^2^Outcome

The study variables were selected according to Fig. 1, which comprise two main pathways that can affect the health outcomes of interest, mortality due to malnutrition and diarrhea in children under 5 years old. The groups of selected causes of mortality were created by aggregating the categories of the International Statistical Classification of Diseases and Health-Related Problems - Tenth revision (ICD-10). Groups A00 - A04 and A06 - A09 were used for mortality due to diarrhea. Those are also classified as Diseases Related to Inadequate Environmental Sanitation (DRSAI) [30]. Categories E40 to E46 were used for mortality due to malnutrition.

### Independent variables and other covariates

Regarding to the independent variables: (i) adequate access to water refers to drinking water from public network, well or individual well; (ii) adequate access to sanitation refers to wastewater collection by sewerage or disposal in a septic tank; (iii) adequate solid waste collection refers to direct collection from the household. PLANSAB [3, 31] and the Joint Monitoring Program (JMP) [32] guidelines were used as references for choosing the independent variables, considering the Brazilian context and the data availability.

For the PBF, two variables were used: proportion of total population covered in the municipality by the Program and proportion of target population (poor and extremely poor families) covered in the municipality by the PBF. Eligible families are those that are classified as poor (per capita monthly income of R$89 to R$178) or extremely poor (per capita monthly income of up to R$89) [12, 14, 15].

Finally, the set of covariables used in this study for controlling confounding effects [22, 33–42] are: (i) per capita monthly income of the municipal population (in Brazilian Reais); (ii) proportion of literate people older than 15 years of age; (iii) proportion of population covered by the Family Health Strategy (ESF), an active door-to-door component of the primary health care policy in the country; (v) proportion of population living in urban areas. The mentioned variables are associated with better wellbeing and access to health services.

### Data source

Data were gathered from the following institutions: (i) Ministry of Health (Mortality Information System/SIM) [43]; (ii) Ministry of Social Development (Social Information Matrix/MIS) [44]; (iii) Brazilian Institute of Geography and Statistics/IBGE (Population censuses of 2000 and 2010, and intercensal estimates) [45] (Table 1).

**Table 1:** Variables available in the information systems and availability period

Variables	Data source/information system	Period
Deaths for diarrhea^a^ (A00 – A04 and A06 – A09)^b^ and for malnutrition (E40 – E46)^b^ and number of children under the age of five	Mortality Information System (SIM) / Informatics Department of the Unified Health System (SUS) (DATASUS)	Years 2006 to 2016
Beneficiary families of the Bolsa Família Program (PBF)	Social Information Matrix (MIS) / Information Evaluation and Management Service (SAGI)	Years 2006 to 2016
Average size of beneficiary families	MIS / SAGI	Years 2007 and 2010
Families eligible for the Bolsa Família Program (PBF)	MIS / SAGI	Years 2006 to 2016
Number of households with coverage for water and sanitation services and number of municipal households	CENSO / IBGE	Years 2000 and 2010^c^
Population exposed to solid waste collection and total municipal population	CENSO / IBGE	Years 2000 and 2010^c^
Per capita monthly income	CENSO/IBGE	Years 2000 and 2010^c^
Proportion of individuals without basic literacy among the population aged 15 and over	CENSO / IBGE	Years 2000 and 2010^c^
Urbanization rate	IBGE	Years 2000 and 2010^c^
Population served by primary care related to the Family Health Strategy (FHS) and total municipal population	Primary Care Information System (SIAB) / DATASUS	Years 2006 to 2016

^a^Only categories related to deaths due to diarrhea were also chosen, which were also classified as Diseases Related to Inadequate Environmental Sanitation (DRSAI) [46]. ^b^International Statistical Classification Codes for Diseases and Health-Related Problems - 10th revision (CID-10). ^c^For variables with information only for the years related to the censuses, 2000 and 2010, interpolation (2006 to 2009) and linear extrapolation (2011 to 2016) methods were applied. For the income variable, due to its non-linear behavior [47], the variation of municipal Gross Domestic Product was used to predict the variation of municipal income and after this procedure, their values were corrected according to the Consumer Price Index Broad (IPCA)

### Data analysis

Descriptive analyses were carried out for the observed municipalities. For statistical inferences, the generalized linear model was adjusted with the Negative Binomial distribution (without and with zero-inflated) to control the overdispersion in the regression models [48–51].

The final regression model was chosen according to the following parameters: (i) Akaike Information Criterion (AIC); (ii) Bayesian Information Criterion (BIC); (iii) better ability to predict the frequency of deaths; (iv) better adjustment of deviations (Deviance and Pearson); (v) possibility of assessing the interactions of interest in the current study.

Most of the study variables were assessed as continuous variables. The variables “Proportion of the target population covered by the PBF” and “access to water” and access to sanitation” were assessed in a categorical manner. The cutoff points used for the categorization were chosen by studying the sample distribution of the values of the variables using the quartiles of the distribution as the main reference, being: (i) proportion of the target population covered by the PBF < 90% (category 0), ≥90 and < 100% (category 1), 100% (category 2); (ii) coverage of access to water: 0 to ≤60% (category 0), > 60% and ≤ 85% (category 1), > 85% and ≤ 100% (category 2); (iii) coverage of access to sanitation: < 20% (category 0), ≥20 and < 50% (category 1), ≥50% and ≤ 100% (category 2).

Also included in the models were terms of interaction between: (i) proportion of population and proportion of target population covered by the PBF and “access to water”; (ii) proportion of population and proportion of target population covered by the PBF and “access to sanitation”; (iii) proportion of population and proportion of target population covered by the PBF and “access to solid waste collection”, in addition to terms of interaction between the variables related to accesses to water, sanitation and solid waste collection.

The significance level of 25% (*p*-value = 0.25) obtained in the univariate analyzes was used as the criterion for selecting variables to compose the multivariate regression models. In these, a significance level of 5% (*p* < 0.05) was used for the variables to remain in the final models. Finally, the significance level of 10% (*p* < 0.10) obtained in the interaction models was used for classifying the observed effects as significant.

Microsoft Office Excel 2010 software was used for the database construction. The R (version 3.0.2) 2013 statistical package (The R Foundation for Statistical Computing) was used for the performing descriptive and inferential analyses.

## Results

### Descriptive statistics

Table 2 shows descriptive statistics for the Brazilian municipalities in relation to variables and period of interest. Among the municipalities, 3526 (out of 5560) presented adequacy of vital statistics [27, 28]. After applying the other inclusion criteria, 3467 municipalities (62.35%) remained in the study. Mortality rates due to malnutrition and diarrhea were reduced between 2006 and 2016 by 64.15 and 41.66%, respectively.

**Table 2:** Descriptive measures of mortality rates by years of study and municipalities selected - Brazil (*N* = 3467)

	2006	2007	2008	2009	2010	2011	2012	2013	2014	2015	2016	Percentage Change 2006-2016
Mortality												
Due to malnutrition	0.53 (2.61)	0.44 (2.52)	0.35 (2.14)	0.36 (2.37)	0.32 (2.38)	0.26 (1.71)	0.28 (2.32)	0.25 (1.85)	0.23 (1.75)	0.25 (1.97)	0.19 (1.43)	- 64.15%
Due to diarrhea	0.12 (1.02)	0.09 (1.02)	0.09 (0.88)	0.07 (0.81)	0.07 (0.75)	0.08 (0.91)	0.07 (0.97)	0.04 (0.52)	0.05 (0.70)	0.07 (1.18)	0.07 (1.11)	- 41.66%
Proportion of coverage of the total population by PBF	31.2% (18.6)	31.1% (19.1)	28.2% (18.0)	30.8% (18.5)	31.2% (19.0)	32.4% (20.6)	33.2% (21.2)	32.4% (21.0)	32.1% (21.8)	30.5% (20.6)	29.3% (20.8)	- 6.09%
Proportion of coverage of the target population by the PBF	87.2% (15.9)	86.8% (15.2)	83.1% (16.5)	90.8% (12.9)	92.3% (12.3)	93.2% (13.1)	94.4% (12.1)	94.3% (12.7)	91.9% (14.6)	91.7% (15.1)	71.7% (34.0)	- 17.77%
Proportion of sanitation coverage	39.3% (29.7)	40.0% (29.7)	40.8% (29.8)	41.5% (30.0)	42.2% (30.3)	43.2% (30.6)	44.3% (30.9)	45.5% (31.2)	46.7% (31.6)	48.0% (31.9)	49.4% (32.3)	25.70%
Proportion of water coverage	87.3% (14.2)	87.2% (14.0)	87.1% (13.9)	87.0% (13.9)	86.9% (14.0)	86.8% (14.1)	86.7% (14.2)	86.6% (14.3)	86.5% (14.5)	86.3% (14.6)	86.2% (14.8)	- 1.26%
Proportion of solid waste collection	63.3% (22.3)	64.8% (21.7)	66.2% (21.3)	67.7% (20.9)	69.2% (20.5)	71.0% (20.3)	73.0% (20.1)	74.9% (19.8)	76.8% (19.6)	78.7% (19.2)	80.6% (18.9)	27.33%
Proportion of coverage of the total population by the FHS	72.2% (31.1)	74.9% (29.7)	79.7% (29.1)	80.5% (28.3)	82.0% (27.6)	83.0% (27.1)	83.3% (26.8)	84.3% (25.3)	86.2% (22.8)	88.3% (20.9)	88.5% (20.9)	22.58%
Urbanization rate (%)	62.6% (21.5)	62.9% (21.3)	63.3% (21.1)	63.6% (21.0)	64.0% (20.9)	64.5% (20.8)	65.1% (20.8)	65.7% (20.8)	66.4% (20.8)	67.0% (20.8)	67.7% (20.9)	8.15%
Per capita monthly income in reais (R$)^a^	353.9 (208.5)	368.2 (218.3)	365.9 (216.9)	357.8 (210.0)	514.4 (259.2)	370.8 (217.8)	369.1 (217.0)	376.2 (222.2)	379.5 (224.1)	396.0 (234.3)	383.0 (226.6)	8.22%
Proportion of literate individuals	71.0% (11.0)	70.9% (10.6)	70.0% (9.9)	68.5% (10.2)	67.0% (10.2)	74.6% (7.0)	73.0% (7.4)	71.4% (7.7)	69.6% (8.1)	67.7% (8.6)	65.6% (9.1)	- 7.61%

Data refer to the mean and (standard deviation). For income^a^, the median was considered. Causes of mortality in children under 5 years old are defined according to the International Classification of Diseases (ICD), 10th revision: diarrheal diseases (A00, A01, A02, A03, A04, A06–08) and malnutrition diseases (E40 - E46). Mortality rates are shown in the table for every 10 thousand children up to 5 years old. *N* Number of municipalities. *PBF* Bolsa Família Program. *FHS* Family Health Strategy

The PBF and access to water coverages showed a decline when comparing the first and last year of the observed period. In turn, the variable related to municipal sanitation coverage increased over the years by 25.70%, when comparing the years 2006 and 2016. However, the coverage was persistently below to 50,00% accross the observed period.

Tables S1 to S5 (supplementary material) show descriptive statistics according to Brazilian regions. The regional distribution of municipalities included in this study is as follow: 1163 (33.54%) from the Northeast, 1037 (29.91%) from the Southeast, 777 (22.42%) from the South, 302 (8.71%) from Midwestern region and 188 (5.42%) from the Northern region. Between 2006 and 2016, all regions showed mortality rates decline due to malnutrition and diarrhea. The highest mortality rates declines were observed in the Southeastern (malnutrition) and Southern (diarrhea) regions.

In the longitudinal observation of the period, the Northern region presented the worst average mortality rates due to malnutrition (0.65/10,000) and diarrhea (0.21/10,000), followed by mortality due to malnutrition in the Northeastern region (0.47/10,000). Regarding the independent variables, Midwestern, Southeastern and Southern regions presented declines in the in the proportion of population covered by the PBF. Midwestern, Northeastern, Southeastern and Southern regions presented declines in the proportion of target population covered by the PBF. All regions showed increased sanitation coverage between the first and last year of studied period. However, only the Southeastern region presented coverage above 75% in 2016. The Northeast was the only region to show an increase in sanitation coverage when comparing the years 2006 and 2016. However, it remained in the last place among regions of the country.

### Inferential statistics

Table 3 shows the estimates of the Incidence Rate Ratios (IRR) and a 95% confidence interval, derived from the adjustments of the multivariate models of Negative Binomial regression with and without zero-inflated, for the average mortality rates due to malnutrition. The results of these models show a negative effect, with an increase in the average mortality rates due to malnutrition, when high coverage of the total municipal population by the PBF is present. Table 4, in turn, shows the IRR and a 95% confidence interval, derived from the adjustments of the multivariate models of Negative Binomial regression with and without zero-inflated, for the average mortality rates due to diarrhea. The results also show a negative effect of the variable coverage of the total population by the PBF, as well as a positive effect, with a decrease in the average mortality rates due to diarrhea, when the target population are highly covered by the PBF in the municipalities (category 1 versus category 0), access to water (categories 1 and 2 versus category 0) and access to sanitation (categories 1 and 2 versus category 0).

**Table 3:** IRR Results - Fixed-effects Negative Binomial (NB) regression model to assess mortality due to malnutrition in children under 5 years old

	NB regression model without zero inflation	NB regression model with zero inflation
	IRR^a^ (CI-95%) *p*-value	IRR^a^ (CI-95%) *p*-value
Bolsa Família Program (PBF) total	1.022 (1.018–1.026) < 2e-16	1.016 (1.011–1.021) 6.04e-11
Literacy population 15 years or older	0.984 (0.978–0.991) 2.71e-06	0.987 (0.980–0.993) 7.54e-05
Population	0.949 (0.912–0.989) 0.00977	0.882 (0.841–0.926) 4.17e-07
Northeast Region	0.512 (0.450–0.584) < 2e-16	0.485 (0.416–0.564) < 2e-16
Midwest Region	1.038 (0.855–1.260) 0.70382	1.975 (1.443–2.702) 2.11e-05
Southeast Region	0.383 (0.322–0.456) < 2e-16	0.342 (0.273–0.427) < 2e-16
South Region	0.381 (0.303–0.476) < 2e-16	0.334 (0.239–0.466) 1.24e-10
Year 2007	0.932 (0.797–1.091) 0.38650	0.908 (0.775–1.063) 0.22289
Year 2008	0.762 (0.644–0.901) 0.00157	0.744 (0.628–0.880) 0.00056
Year 2009	0.670 (0.564–0.794) 4.35e-06	0.657 (0.554–0.780) 1.64e-06
Year 2010	0.571 (0.478–0.681) 6.91e-10	0.561 (0.469–0.671) 2.13e-10
Year 2011	0.566 (0.468–0.683) 4.09e-09	0.560 (0.463–0.677) 2.14e-09
Year 2012	0.452 (0.370–0.551) 6.52e-15	0.444 (0.364–0.543) 2.01e-15
Year 2013	0.454 (0.372–0.552) 4.28e-15	0.457 (0.375–0.557) 8.29e-15
Year 2014	0.440 (0.360–0.535) 3.69e-16	0.438 (0.359–0.534) 3.71e-16
Year 2015	0.408 (0.332–0.499) < 2e-16	0.403 (0.328–0.494) < 2e-16
Year 2016	0.373 (0.302–0.459) < 2e-16	0.378 (0.306–0.466) < 2e-16

Model without zero inflation: AIC: 15248. BIC: 15429.37. 2 x loglik: - 15,210,437. Zero inflation model: AIC: 15190. BIC: 15420. 2 x log-lik: - 15,134

The comparison references of the models refer to the regions: North region and for the year: 2006

^a^Ratio for incidence rates

**Table 4:** IRR Results - Fixed-effects Negative Binomial (NB) regression model to assess mortality due to diarrhea in children under 5 years old

	NB regression model without zero inflation	NB regression model with zero inflation
	IRR^a^ (CI-95%) *p*-value	IRR^a^ (CI-95%) *p*-value
Bolsa Família Program (PBF) target		
≥ 90% ≤ 99.9% (category 1)	0.7058 (0.5427–0.9113) 0.007981	0.7363 (0.5591–0.9695) 0.029240
100% (category 2)	0.8203 (0.6562–1.0248) 0.080057	0.9039 (0.7165–1.1404) 0.394133
Bolsa Família Program (PBF) total	1.0220 (1.0118–1.0322) 1.71e-05	1.0218 (1.0117–1.0320) 2.20e-05
Environmental Health		
Access to water		
> 60% ≤ 85% (category 1)	0.6235 (0.4666–0.8399) 0.001897	0.6267 (0.4678–0.8396) 0.001737
> 85% ≤ 100% (category 2)	0.5779 (0.4048–0.8221) 0.002627	0.5946 (0.4189–0.8440) 0.003624
Access to sanitation		
≥ 20 < 50% (category 1)	0.7927 (0.6330–0.9894) 0.040499	0.7579 (0.5912–0.9716) 0.028718
≥ 50 < 100% (category 2)	0.8501 (0.6317–1.1416) 0.277095	0.7152 (0.5106–1.0017) 0.051177
Solid waste collection	0.9901 (0.9812–0.9991) 0.030414	0.9902 (0.9812–0.9993) 0.035712
Family Health Strategy (FHS)	0.9980 (0.9946–1.0014) 0.237284	0.9952 (0.9915–0.9989) 0.011344
Literacy population 15 years or older	0.9783 (0.9655–0.9914) 0.001163	0.9711 (0.9574–0.9850) 5.32e-05
Per capita income	0.7943 (0.5924–1.0666) 0.118250	0.6342 (0.4582–0.8776) 0.006007
Urbanization rate	1.0159 (1.0072–1.0248) 0.000319	1.0266 (1.0164–1.0369) 2.42e-07
Northeast Region	0.3916 (0.3021–0.5094) 2.39e-12	0.4428 (0.3367–0.5824) 5.69e-09
Midwest Region	1.0266 (0.6933–1.5124) 0.892349	3.0768 (1.7230–5.4945) 0.000145
Southeast Region	0.4497 (0.3134–0.6450) 9.49e-06	0.4999 (0.3439–0.7269) 0.000283
South Region	0.7451 (0.4937–1.1185) 0.150701	0.8881 (0.5809–1.3575) 0.583517
Year 2007	0.8551 (0.6222–1.1728) 0.331941	0.8528 (0.6228–1.1676) 0.320511
Year 2008	0.9136 (0.6650–1.2534) 0.576897	0.8999 (0.6569–1.2326) 0.511081
Year 2009	0.6731 (0.4747–0.9489) 0.024212	0.6729 (0.4768–0.9498) 0.024283
Year 2010	0.7823 (0.5401–1.1289) 0.190924	0.8082 (0.5603–1.1658) 0.254591
Year 2011	0.7859 (0.5405–1.1362) 0.197112	0.7646 (0.5280–1.1073) 0.155411
Year 2012	0.5942 (0.3984–0.8779) 0.009309	0.5859 (0.3952–0.8686) 0.007778
Year 2013	0.5577 (0.3712–0.8294) 0.004322	0.5424 (0.3628–0.8110) 0.002877
Year 2014	0.5122 (0.3356–0.7714) 0.001381	0.5077 (0.3353–0.7690) 0.001372
Year 2015	0.5507 (0.3610–0.8289) 0.004266	0.5439 (0.3588–0.8247) 0.004133
Year 2016	0.5837 (0.3862–0.8735) 0.009270	0.5719 (0.3798–0.8611) 0.007449

Model without zero inflation: AIC: 5772.70. BIC: 6012.05. 2 x loglik: - 5716,685. Zero inflation model: AIC: 5746.00. BIC: 6105.05. 2 x loglik: - 5662.0

The comparison references of the models refer to the target PBF: < 90% (category 0); access to water: ≤ 60% (category 0); sanitation: < 20% (category 0); regions: North region and for the year: 2006

^a^Ratio for incidence rates

Table 5 provides answers to the main question that served as the basis for this study. It presents the estimates of the IRR, derived from the adjustments of the multivariate models of Negative Binomial regression with and without zero-inflated, including the interaction terms significant between access to PBF and to environmental sanitation services. For the outcome of mortality due to malnutrition, the interactions between the variables were significant: (i) proportion of total population covered by the PBF and collection of solid waste; (ii) proportion of total population covered by the PBF and access to sanitation (category 1); (iii) proportion of total population covered by the PBF and access to sanitation (category 2). In turn, for the outcome of mortality due to diarrhea, the interactions between the variables were significant: (i) proportion of total population covered by the PBF and access to water (category 1); (ii) proportion of total population covered by the PBF and access to sanitation (category 2); (iii) proportion of target population covered by the PBF (category 2) and access to water (category 2); (iv) coverage of the access to water (category 1 and 2) and collection of solid waste.

**Table 5:** Results of the Fixed-effects Negative Binomial (NB) regression model to assess the interaction to the outcome of mortality due to malnutrition and diarrhea in children under 5years old

	NB regression model without zero inflation Interactions	NB regression model with zero inflation Interactions
	IRR^a^ (CI-90%) *p*-value	IRR^a^ (CI-90%) *p*-value
**Malnutrition**		
Interaction between: PBF total Solid waste collection^#^	1.0004 (1.0002–1.0005) 2.56e-09	..
Interaction between: PBF total Access to sanitation^#^ ≥ 20 < 50% (category 1)	..	1.0061 (0.9991–1.0132) 0.088284
Interaction between: PBF total Access to sanitation^#^ ≥ 50 < 100% (category 2)	..	1.0073 (1.0002–1.0145) 0.045116
**Diarrhea**		
Interaction between: PBF total Access to water > 60% ≤85% (category 1)	0.9774658 (0.9533472–1.002195) 0.073767	0.9780299 (0.9549282–1.001691) 0.068526
Interaction between: 100% PBF target (category 2) Access to water > 85% ≤ 100% (category 2)	0.5463344 (0.285883–1.044068) 0.067326	0.558559 (0.2980557–1.046745) 0.069151
Interaction between: PBF total Access to sanitation ≥50 < 100% (category 2)	..	0.9892611 (0.9764852–1.002204) 0.103500
Interaction between: Access to water > 60% ≤ 85% (category 1) Solid waste collection	0.9837505 (0.9672618–1.00052) 0.057466	0.9892611 (0.9764852–1.002204) 0.080497
Interaction between: Access to water > 85% ≤ 100% (category 2) Solid waste collection	0.9739308 (0.9581343–0.9899877) 0.001545	0.9746869 (0.9582469–0.991409) 0.003134

Malnutrition: The model comparison references refer to sanitation: < 20% (category 0)

^#^The variables: solid waste collection, sanitation and access to water were not significant in multivariate analyzes. However, solid waste collection and sanitation were significant in the interaction models, according to the values presented in this Table. In the interaction models, their individual values were for: solid waste collection (IRR 0.9815 CI 0.9750–0.9880 *p*-value = 2.93e-08), for sanitation category 1 (IRR 0.7611 CI 0.5316–1.0897 *p*-value = 0.136025) and for sanitation category 2 (IRR 0.7520 CI 0.5314–1.0640 *p*-value = 0.107465)

Diarrhea: The comparison references of the models refer to the target PBF: < 90% (category 0); access to water: ≤ 60% (category 0); sanitation: < 20% (category 0)

^a^Ratio for incidence rates ..interactions were not possible to be adjusted

The effects identified were statistically significant even after adjusting the regression models, considering the zero-inflated related to outcomes of interest.

## Discussion

The results of the study show that average mortality rates due to diarrhea and malnutrition in children younger than 5 years old, decreased when comparing the years 2006 and 2016, in the observed Brazilian municipalities. The highest mortality declines due to diarrhea occurred in the Southern region, followed by the Midwestern region. The highest mortality declines due to malnutrition occurred in the Southeastern region.

In contrast, longitudinal analysis of the data shows a concentration of higher average mortality rates due to malnutrition in the Northern, Northeastern and Midwestern regions and due to diarrhea in the Northern region. The results are consistent with the study performed in 2018 by Risse et al. [52].

Improvements in the health conditions of the population can be observed when comparing the results of the current study with others that found average mortality rates due to malnutrition and diarrhea in 2009 [22] and from 2000 to 2015 [53] higher than the results found in the current study. The results of the Binomial Negative regression models suggest a protective effect of the population living in the Northeastern, Southeastern and Southern regions, when compared to the Northern region of the country. However, the regional differences remain relevant disfavoring Northern, Northeastern and Midwestern regions. That illustrates the need for public policies tailored for reducing health disparities among Brazilian regions and municipalities.

Between 2006 and 2016, PBF coverage declined for both total population and target population. In the beginning of the period, it was observed a good match between high PBF coverage and high proportion of poorer populations. Subsequent reductions on PBF coverage suggest positive impact of the program on poverty reduction. After 2014, however, successive reductions on PBF coverage of the target population, from 91.4% (2014) to 71.7% (2016, the lowest coverage), indicate reduction to access by families in situations of social and economic vulnerability. That can result in setbacks in the improvements for poverty reduction and in incentives for using preventive health care, specially the access to the Family Health Strategy (FHS), with possible consequences to mortality rates. The FHS, an important gateway to primary care in the country, presenting over 85% coverage since 2014 [54, 55], provides compliance with one of the conditionalities of the PBF. Studies have shown PBF health conditionality to result in positive impacts in morbimortality reduction of malnutrition and diarrhea [22].

Still in relation to the decrease in municipal coverage, the education variable showed proportions of literacy in decline over the years of analysis, contrary to expectations [22, 56–58]. Also, the proportion of households with access to water reduced over the period. That is probably associated with difficulties on maintaining the investment for holding the high coverage existing at the beginning of the period.

Between 2006 and 2016, sanitation coverage increased in all years and regions. However, those coverages have never reached 50% for the whole country. The high degree of heterogeneity in sanitation coverage has been presented over the period: 15.9% (Northern region) to 67.7% (Southeastern region) in 2006 and 25.3% (Northern region) to 75.2% (Southeastern region) in 2016. In turn, the variables: proportion of urbanized population, collection of solid waste and municipal per capita income, showed consistent increase between 2006 and 2016, which may be associated with social improvements.

The multivariate model related to mortality due to diarrhea (Table 4) shows results that highlight the importance of high coverage of the target population by the PBF and adequate conditions of access to water, sanitation and solid waste collection in reducing mortality rates due to this disease. In relation to these determinants, studies indicate favorable results of access to conditional cash transfer programs (including PBF), directly reflecting on the improvement in the beneficiary family income and in better health and nutrition conditions [22, 54, 59–70]. Other studies point out how access/non-access to drinking water and sanitation has a strong impact on health and disease processes, whether in the outcomes related to infectious diseases, such as diarrhea, or in the nutritional status of the exposed population [10, 37, 61, 71–83]. In this sense, these policies are important and complementary for improving the quality of life and providing safe environments for child growth and development. However, although the effects of interventions in environmental health and PBF have been addressed in these studies, none of them have assessed the effect of simultaneous presence of the two public policies, relating them to the outcomes mortality by diarrhea and malnutrition.

In this study, the choice of the timeframe for evaluating the effects of public policies, PBF and environmental health, allowed for the exploration of their joint effects. In 2003, the PBF started its activities. It was only in 2006 that PBF consolidated data related to municipal coverage and the target population for the whole country. In 2007, water, sanitation and waste management were regulated by means of Law 11.455 [84], establishing national guidelines for basic environmental health in Brazil.

For mortality due to malnutrition, the interaction models (Table 5) show that the variables of access to sanitation (categories 1 and 2 x category 0) and collection of solid waste modified the effect of the variable coverage of the total population by PBF. The results from Table 5 indicate that in very poor municipalities access to sanitation and solid waste collection combined with elevated PBF coverage are not sufficient to reduce average rates of malnutrition mortality.

For mortality due to diarrhea (Table 5), the variables of access to water (category 1 x category 0) and access to sanitation (category 2 x category 0) modified the effect of the variable coverage of the total population by PBF, resulting in the inversion of the ratio of the average mortality rates observed when only the variable coverage of the total population by the PBF is present. When the interaction of the PBF coverage is analyzed in the target population (category 2 x category 0) and access to water (category 2 x category 0) this expected effect is observed. The results suggest that municipalities that combine 100% coverage of the target population by the PBF with coverage of access to water above 85% have lower average rates of mortality due to diarrhea than municipalities not exposed to those levels of coverage. Finally, there is a change in the effect of the access to water variable (categories 1 and 2 x category 0) and the collection of solid waste variable, with a decrease in the average mortality rates due to diarrhea. In view of the above, the interaction models used indicated, for the outcome of diarrhea mortality, the simultaneous presence of better coverage of the PBF and access to water and sanitation resulting in decrease in the average mortality rates due to diarrhea, validate the hypothesis of their joint beneficial effects in relation to diarrhea mortality (Fig. 1). (insert Fig. 1).

Several studies have observed that the contribution of socioeconomic variables to the improvement of wellbeing and the subsequent impact on the health status of the poor and extremely vulnerable population groups [21, 22, 33, 38, 54, 66]. For large Brazilian cities and poor population groups in general, the growth in formal employment and the minimum wage produced a significant impact reducing socioeconomic and health inequalities [22]. For small cities and extremely poor population groups, social benefits played a larger role because those municipalities are away from the dynamism of the major economic centers. Therefore, they have been unable to produce enough jobs for reducing structural unemployment and meeting the demands of younger workers. The extremely poor population groups from both big and small cities have very little access to the formal job market, being the last to be hired and first to be sacked from their low-qualified jobs [22]. In this study, the potential confounding variables were treated and controlled for in the regression models, resulting in responses that indicated that the observed changes occurred possibly due to the increase in coverage of the target population by the Bolsa Família Program and environmental sanitation services.

### Strengths and limitations of the study

The main limitations of the current study refer to the use of secondary data and the need to apply interpolation and linear extrapolation methods to estimate annual values for some variables. However, any potential bias, which could have caused a decrease in the real fluctuations of the measures over the years, was minimized by comparing the interpolated and extrapolated data with the real data collected through PNADs [85] and SNIS [86], and verifying compatible measures for these values. The current study has some built-in characteristics that reinforce the validity of its findings. Firstly, only municipalities that presented adequacy of vital statistics data were included, which ensures that the information collected related to mortality due to malnutrition and diarrhea is reliable, increasing its internal validity. Secondly, due to the use of the variable coverage of the target population by the PBF, which is specifically targeted to families in situations of social vulnerability, it was possible to analyze the chances of this group being affected by processes of illness and death due these conditions. In addition, one of the strengths of the study was the possibility, through specific statistical analysis, of assessing all 3467 municipalities participating in the research including 38.137 observations. Even with the zero-inflated characteristic of the response variables, the results reflect the reality of 62.35% of Brazilian municipalities. Finally, it was possible to obtain stronger evidence related to the significance of the variables due to the use of participant panel data and longitudinal analysis of the data when compared to studies using only cross-sectional data [87]. Through a mixed ecological study it was possible to analyze broad socio-environmental contexts, information not collected at the individual level [88]. The Negative Binomial generalized linear model of fixed effects with and without inflated-zero modeling was utilized quite effectively, and although it forecasted a number of cases of deaths due to malnutrition and diarrhea slightly lower than the actual sample data (losses of 8.07% due to malnutrition and 0.24% due to diarrhea), it allowed for an assessment of the significance of the exposure variables.

## Conclusion

In view of the presented results, the maintenance of the PBF, with full coverage of the target population, combined with universal environmental health policies, for all Brazilian municipalities and with greater attention given to the Northern, Northeastern and Midwestern regions, should be a government priority, as this will provide enhanced beneficial effects on child health. The maintenance and expansion of these programs requires prioritization and planning by the country’s federal government to meet the demand, along with municipal authorities, in adapting their health, education and housing structures, in order to allow the beneficiary to be assisted and to comply with the Program’s conditionalities.

There is a clear downward trend in the main social protection variables, especially in 2016, indicating need for: (i) the PBF target population is fully covered; (ii) universal public environmental health policies; (iii) increased in the literacy of people aged 15 years and old; (iv) increased population coverage by the FHS, through the strengthening of the Single Health System (SUS), thus allowing for: the construction of safer environments, children survival and the improvement on life quality development.

From a global point of view, the findings of this study indicate the importance of associating different social programs when focusing on combating poverty. Systemic interventions, which at the same time aim at protecting individual or families from poverty associated with precarious environmental conditions, are fundamental for eliminating routes of transmission of infectious and parasitic diseases, reducing child deaths.

## Supplementary Information

**Additional file 1: Table S1.** Descriptive measures of mortality rates by years of study and municipalities selected – Region Midwest (*N* = 302). **Table S2**. Descriptive measures of mortality rates by years of study and municipalities selected – Region Northeast (*N* = 1.163). **Table S3**. Descriptive measures of mortality rates by years of study and municipalities selected – Region North (*N* = 188). **Table S4**. Descriptive measures of mortality rates by years of study and municipalities selected – Region Southeast (*N* = 1.037). **Table S5**. Descriptive measures of mortality rates by years of study and municipalities selected – Region South (*N* = 777).

## Data Availability

The study was carried out using publicly available data, available in the information systems of the Ministry of Health (MS) [41], Ministry of Social Development (MDS) [42] and Brazilian Institute of Geography and Statistics (IBGE) [43] reported in the methods section and data and collection procedures subsection. Most of the data generated and analyzed during this study are included in the body of the manuscript and as supplementary material. Information from data not shown in a table and referenced in the text of the manuscript is publicly accessible in the information systems mentioned above. All the data used in the research, as well as the database produced by the researchers are with the corresponding author and other authors of the manuscript, in addition to being on the platform of the René Rachou Institute, Fiocruz Minas and may be released as needed and upon reasonable request. Links: MS: http://tabnet.datasus.gov.br/cgi/menu_tabnet_php.htm MDS: http://mds.gov.br/assuntos/bolsa-familia IBGE: https://www.ibge.gov.br/

## Abbreviations

AIC: Akaike Information Criterion
BIC: Bayesian Information Criterion
CadÚnico: Single Social Registry of the Federal Government Programs
CCT: Conditional Cash Transfer Program
CID-10: Statistical International Classification Code of Diseases and Health-Related Problems - 10th revision
DATASUS: Department of Informatics of the Single Health System
DRSAI: Diseases Related to Inadequate Environmental Sanitation
FHS: Family Health Strategy
GDP: Gross Domestic Product
IBGE: Brazilian Institute of Geography and Statistics
IPCA: National Extended Consumer Price Index
IRR: Ratio for Incidence Rates
JMP: *Joint Monitoring Program*
MIS: Social Information Matrix
PBF: Bolsa Família Program
PLANSAB: Basic National Sanitation Plan
PNAD: National Household Sample Survey
SIM: Mortality Information System
SMR: Age-Standardized Mortality Rate
SNIS: National Sanitation Information System
