# Supplementary material for: Combination of conditional cash transfer program and environmental health interventions reduces child mortality: an ecological study of Brazilian municipalities
Source: BMC Public Health. 2021 Mar 31;21:627. doi: 10.1186/s12889-021-10649-4 (PMC8011115; doi:10.1186/s12889-021-10649-4)
Supplement: Supplementary file 1 — Additional file 1: Table S1. Descriptive measures of mortality rates by years of study and municipalities selected – Region Midwest (N = 302). Table S2. Descriptive measures of mortality rates by years of study and municipalities selected – Region Northeast (N = 1.163). Table S3. Descriptive measures of mortality rates by years of study and municipalities selected – Region North (N = 188). Table S4. Descriptive measures of mortality rates by years of study and municipalities selected – Region Southeast (N = 1.037). Table S5. Descriptive measures of mortality rates by years of study and municipalities selected – Region South (N = 777). [file 12889_2021_10649_MOESM1_ESM.docx]

SUPPLEMENTARY MATERIAL

Table S1: Descriptive measures of mortality rates by years of study and municipalities selected – Region Midwest (N=302)

|  | **2006** | **2007** | **2008** | **2009** | **2010** | **2011** | **2012** | **2013** | **2014** | **2015** | **2016** | **Percentage Change 2006-2016** |
| --- | --- | --- | --- | --- | --- | --- | --- | --- | --- | --- | --- | --- |
|  |  |  |  |  |  |  |  |  |  |  |  |  |
| Mortality |  |  |  |  |  |  |  |  |  |  |  |  |
| Due to malnutrition | 0.38 (2.29) | 0.49 (3.29) | 0.24 (1.35) | 0.42 (2.60) | 0.34 (2.94) | 0.26 (1.94) | 0.37 (2.37) | 0.13 (0.91) | 0.38 (2.40) | 0.46 (2.84) | 0.23 (1.68) | - 39.47% |
| Due to diarrhea | 0.08 (0.87) | 0.04 (0.44) | 0.11 (0.80) | 0.08 (0.69) | 0.07 (0.58) | 0.10 (1.03) | 0.18 (2.49) | 0.04 (0.51) | 0.03 (0.35) | 0.10 (1.06) | 0.03 (0.38) | - 62.50% |
| Proportion of coverage of the total population by PBF | 27.1% (8.1) | 28.2% (18.0) | 18.8% (6.9) | 22.1% (7.5) | 22.5% (7.6) | 22.5% (8.0) | 23.2% (8.3) | 22.4% (8.5) | 21.9% (9.0) | 20.8% (8.6) | 19.3% (8.4) | - 28.78% |
| Proportion of coverage of the target population by the PBF | 81.5% (19.8) | 83.1% (16.5) | 78.7% (19.3) | 90.6% (12.9) | 92.5% (11.7) | 93.0% (12.0) | 94.7% (11.0) | 95.3% (11.1) | 93.4% (13.0) | 93.0% (13.4) | 70.7% (34.0) | - 13.25% |
| Proportion of sanitation coverage | 18.9% (17.5) | 20.7% (18.2) | 21.2% (19.0) | 22.4% (20.0) | 23.5% (21.0) | 25.1% (22.3) | 26.7% (23.8) | 28.8% (25.5) | 30.8% (27.1) | 32.8% (28.6) | 35.0% (30.2) | 85.18% |
| Proportion of water coverage | 95.4% (4.7) | 95.1% (5.1) | 94.7% (5.5) | 94.3% (6.0) | 94.0% (6.4) | 93.7% (6.8) | 93.3% (7.2) | 93.0% (7.6) | 92.7% (8.0) | 92.4% (8.3) | 92.1% (8.6) | - 3.45% |
| Proportion of solid waste collection | 70.3% (15.1) | 66.2% (21.3) | 72.6% (14.5) | 73.8% (14.2) | 75.0% (14.1) | 76.3% (14.0) | 77.8% (14.0) | 79.3% (14.0) | 80.9% (14.0) | 82.5% (14.1) | 84.0% (14.2) | 19.48% |
| Proportion of coverage of the total population by the FHS | 77.4% (23.4) | 79.7% (29.1) | 84.7% (21.5) | 84.9% (20.6) | 86.4% (19.8) | 87.2% (19.0) | 87.4% (18.6) | 88.0% (17.9) | 88.1% (17.0) | 89.6% (16.4) | 90.0% (16.3) | 16.27% |
| Urbanization rate (%) | 72.3% (15.8) | 63.3% (21.1) | 72.9% (15.6) | 73.1% (15.5) | 73.4% (15.5) | 74.0% (15.5) | 74.5% (15.7) | 75.1% (15.8) | 75.6% (16.0) | 76.3% (16.1) | 76.9% (16.3) | 6.36% |
| Per capita monthly income in reais (R$)* | 432.7 (164.2) | 449.3 (216.9) | 447.3 (173.7) | 433.2 (171.1) | 605.9 (173.3) | 453.9 (175.7) | 451.1 (178.6) | 458.7 (182.3) | 460.5 (185.0) | 481.0 (194.6) | 470.2 (190.4) | 8.66% |
| Proportion of literate individuals | 73.0% (6.8) | 72.8% (6.9) | 72.3% (5.3) | 71.0% (5.6) | 69.2% (6.3) | 76.4% (4.9) | 75.0% (5.2) | 73.5% (5.7) | 61.9% (6.1) | 70.2% (6.6) | 68.4% (7.1) | - 6.30% |
| Data refer to the mean and (standard deviation). For income*, the median was considered. Causes of mortality in children under five years old are defined according to the International Classification of Diseases (ICD), 10th revision: diarrheal diseases (A00, A01, A02, A03, A04, A06-08) and malnutrition diseases (E40 - E46). Mortality rates are shown in the table for every ten thousand children up to five years old. N = number of municipalities. PBF=Bolsa Família Program. FHS=Family Health Strategy. | | | | | | | | | | | | |
|  |  |  |  |  |  |  |  |  |  |  |  |  |

Table S2: Descriptive measures of mortality rates by years of study and municipalities selected – Region Northeast (N=1.163)

|  | **2006** | **2007** | **2008** | **2009** | **2010** | **2011** | **2012** | **2013** | **2014** | **2015** | **2016** | **Percentage Change**  **2006-2016** |
| --- | --- | --- | --- | --- | --- | --- | --- | --- | --- | --- | --- | --- |
|  |  |  |  |  |  |  |  |  |  |  |  |  |
| Mortality |  |  |  |  |  |  |  |  |  |  |  |  |
| Due to malnutrition | 0.77 (2.45) | 0.70 (2.38) | 0.60 (2.41) | 0.54 (2.23) | 0.44 (2.08) | 0.36 (1.76) | 0.35 (2.09) | 0.46 (2.47) | 0.32 (1.93) | 0.31 (1.74) | 0.32 (1.95) | - 58.44% |
| Due to diarrhea | 0.20 (1.24) | 0.11 (0.79) | 0.14 (1.20) | 0.08 (0.81) | 0.08 (0.74) | 0.08 (0.71) | 0.08 (0.77) | 0.06 (0.64) | 0.08 (0.90) | 0.10 (1.31) | 0.08 (0.85) | - 60.00% |
| Proportion of coverage of the total population by PBF | 52.1% 11.4) | 52.7% (10.8) | 48.9% (10.0) | 51.9% (9.4) | 52.9% (9.6) | 56.1% (10.0) | 57.4% (10.4) | 56.3% (10.2) | 56.9% (11.2) | 53.9% (10.5) | 52.8% (11.2) | 1.34% |
| Proportion of coverage of the target population by the PBF | 88.4% (11.9) | 89.6% (10.7) | 88.3% (10.9) | 94.4% (7.1) | 96.2% (6.0) | 98.8% (3.8) | 99.2% (3.1) | 99.5% (2.4) | 99.2% (3.3) | 99.1% (3.6) | 79.5% (29.1) | - 10.06% |
| Proportion of sanitation coverage | 22.5% (19.4) | 23.1% (19.7) | 23.7% (20.2) | 24.2% (20.8) | 24.8% (21.5) | 25.7% (22.2) | 26.8% (23.0) | 28.0% (24.0) | 29.3% (24.9) | 30.7% (25.9) | 32.2% (27.1) | 43.11% |
| Proportion of water coverage | 74.4% (16.0) | 74.7% (15.9) | 75.0% (15.9) | 75.2% (16.0) | 75.5% (16.3) | 75.8% (16.5) | 76.1% (16.8) | 76.4% (17.1) | 76.6% (17.5) | 76.9% (16.9) | 77.2% (18.2) | 3.76% |
| Proportion of solid waste collection | 48.6% (19.7) | 50.3% (19.5) | 52.1% (19.4) | 53.9% (19.3) | 55.6% (19.3) | 57.7% (19.3) | 60.0% (19.4) | 62.3% (19.5) | 64.8% (19.7) | 67.3% (19.9) | 69.9% (20.0) | 43.82% |
| Proportion of coverage of the total population by the FHS | 80.7% (24.0) | 84.6% (21.2) | 90.5% (18.0) | 91.6% (16.3) | 92.9% (15.0) | 93.5% (14.2) | 93.4% (13.8) | 93.9% (12.7) | 95.6% (10.7) | 96.1% (10.0) | 96.2% (9.9) | 19.20% |
| Urbanization rate (%) | 53.4% (16.7) | 53.7% (18.6) | 54.1% (18.5) | 54.5% (18.4) | 54.9% (18.4) | 55.4% (18.4) | 56.0% (18.5) | 56.7% (18.6) | 57.4% (18.6) | 58.1% (18.8) | 58.8% (18.9) | 10.11% |
| Per capita monthly income in reais (R$)* | 168.5 (73.3) | 173.0 (76.1) | 172.8 (75.7) | 171.0 (72.6) | 273.0 (100.9) | 177.4 (74.0) | 176.3 (73.6) | 179.7 (75.7) | 182.4 (80.4) | 190.5 (87.0) | 183.6 (84.0) | 8.96% |
| Proportion of literate individuals | 61.0% (8.9) | 61.5% (8.8) | 60.% (7.8) | 59.4% (8.0) | 58.2% (7.9) | 69.3% (5.8) | 67.9% (6.1) | 66.4% (6.5) | 64.8% (6.8) | 63.1% (7.2) | 61.3% (7.7) | 0.49% |
| Data refer to the mean and (standard deviation). For income*, the median was considered. Causes of mortality in children under five years old are defined according to the International Classification of Diseases (ICD), 10th revision: diarrheal diseases (A00, A01, A02, A03, A04, A06-08) and malnutrition diseases (E40 - E46). Mortality rates are shown in the table for every ten thousand children up to five years old. N = number of municipalities. PBF=Bolsa Família Program. FHS=Family Health Strategy. | | | | | | | | | | | | |
|  |  |  |  |  |  |  |  |  |  |  |  |  |

Table S3: Descriptive measures of mortality rates by years of study and municipalities selected – Region North (N=188)

|  | **2006** | **2007** | **2008** | **2009** | **2010** | **2011** | **2012** | **2013** | **2014** | **2015** | **2016** | **Percentage Change 2006-2016** |
| --- | --- | --- | --- | --- | --- | --- | --- | --- | --- | --- | --- | --- |
|  |  |  |  |  |  |  |  |  |  |  |  |  |
| Mortality |  |  |  |  |  |  |  |  |  |  |  |  |
| Due to malnutrition | 0.96 (3.92) | 0.82 (2.34) | 0.65 (2.25) | 0.84 (2.79) | 0.37 (1.20) | 0.81 (3.17) | 0.74 (3.68) | 0.62 (2.24) | 0.39 (1.80) | 0.43 (1.69) | 0.52 (1.71) | - 45.83% |
| Due to diarrhea | 0.44 (1.93) | 0.17 (0.83) | 0.21 (1.05) | 0.25 (1.40) | 0.24 (1.11) | 0.23 (1.46) | 0.17 (1.28) | 0.16 (0.94) | 0.10 (0.82) | 0.07 (0.67) | 0.27 (1.83) | - 38.63% |
| Proportion of coverage of the total population by PBF | 35.2% (11.4) | 38.6% (10.7) | 36.0% (10.5) | 40.3% (10.8) | 41.2% (11.8) | 44.0% (14.3) | 46.0% (14.6) | 46.2% (15.3) | 45.8% (16.2) | 43.9% (15.9) | 42.6% (17.9) | 21.02% |
| Proportion of coverage of the target population by the PBF | 69.7% (18.7) | 74.2% (15.4) | 73.7% (15.0) | 85.6% (12.3) | 88.1% (11.4) | 92.8% (11.2) | 95.7% (8.6) | 96.9% (7.8) | 95.6% (9.2) | 95.3% (10.0) | 74.9% (31.1) | 7.46% |
| Proportion of sanitation coverage | 15.9% (12.5) | 16.4% (12.7) | 16.9% (13.0) | 17.4% (13.5) | 17.9% (14.3) | 18.7% (15.2) | 19.8% (16.4) | 21.0% (17.9) | 22.3% (19.5) | 23.7% (21.2) | 25.3% (22.8) | 59.11% |
| Proportion of water coverage | 88.0% (11.7) | 87.8% (11.8) | 87.5% (12.0) | 87.3% (12.3) | 87.0% (12.6) | 86.8% (12.8) | 86.6% (13.1) | 86.5% (13.3) | 86.3% (13.6) | 86.1% (13.8) | 86.0% (14.1) | - 2.27% |
| Proportion of solid waste collection | 49.2% (19.1) | 51.3% (18.8) | 53.5% (18.5) | 55.7% (18.3) | 57.8% (18.2) | 60.4% (18.3) | 63.1% (18.5) | 66.1% (18.9) | 69.2% (19.3) | 72.3% (19.8) | 75.2% (19.9) | 52.84% |
| Proportion of coverage of the total population by the FHS | 64.8% (30.9) | 72.3% (29.9) | 77.5% (27.3) | 79.5% (25.5) | 82.3% (24.1) | 83.2% (23.7) | 82.3% (24.2) | 82.5% (23.7) | 86.1% (20.6) | 88.6% (18.4) | 89.7% (17.9) | 38.42% |
| Urbanization rate (%) | 58.9% (18.5) | 59.2% (19.4) | 59.4% (19.3) | 59.7% (19.3) | 60.0% (19.2) | 60.5% (19.3) | 61.1% (19.4) | 61.8% (19.5) | 62.6% (19.6) | 63.3% (19.8) | 64.0% (19.9) | 8.65% |
| Per capita monthly income in reais (R$)* | 235.9 (113.9) | 246.4 (120.6) | 245.5 (120.2) | 238.0 (109.6) | 350.9 (155.0) | 240.3 (113.8) | 237.3 (113.6) | 237.4 (113.9) | 239.8 (116.1) | 248.1 (122.3) | 236.3 (118.7) | 0.16% |
| Proportion of literate individuals | 68.0% (10.2) | 68.6% (9.9) | 67.4% (8.7) | 66.1% (8.9) | 65.3% (8.6) | 76.0% (5.4) | 74.7% (5.7) | 73.3% (6.1) | 71.8% (6.5) | 70.3% (6.9) | 68.7% (7.4) | 1.02% |
| Data refer to the mean and (standard deviation). For income*, the median was considered. Causes of mortality in children under five years old are defined according to the International Classification of Diseases (ICD), 10th revision: diarrheal diseases (A00, A01, A02, A03, A04, A06-08) and malnutrition diseases (E40 - E46). Mortality rates are shown in the table for every ten thousand children up to five years old. N = number of municipalities. PBF=Bolsa Família Program. FHS=Family Health Strategy. | | | | | | | | | | | | |
|  |  |  |  |  |  |  |  |  |  |  |  |  |

Table S4: Descriptive measures of mortality rates by years of study and municipalities selected – Region Southeast (N=1.037)

|  | **2006** | **2007** | **2008** | **2009** | **2010** | **2011** | **2012** | **2013** | **2014** | **2015** | **2016** | **Percentage Change 2006-2016** |
| --- | --- | --- | --- | --- | --- | --- | --- | --- | --- | --- | --- | --- |
|  |  |  |  |  |  |  |  |  |  |  |  |  |
| Mortality |  |  |  |  |  |  |  |  |  |  |  |  |
| Due to malnutrition | 0.40 (2.76) | 0.15 (1.16) | 0.08 (0.71) | 0.12 (1.36) | 0.21 (1.77) | 0.17 (1.46) | 0.28 (2.95) | 0.15 (1.83) | 0.15 (1.67) | 0.23 (2.49) | 0.05 (0.59) | - 87.50% |
| Due to diarrhea | 0.05 (0.73) | 0.09 (1.33) | 0.02 (0.40) | 0.02 (0.26) | 0.04 (0.86) | 0.04 (0.63) | 0.02 (0.30) | 0.02 (0.32) | 0.04 (0.69) | 0.06 (1.48) | 0.03 (0.55) | - 40.00% |
| Proportion of coverage of the total population by PBF | 20.4% (10.8) | 19.8% (11.0) | 17.2% (10.0) | 19.1% (9.8) | 19.3% (10.0) | 19.4% (19.5) | 20.0% (11.0) | 19.4% (11.0) | 18.4% (11.2) | 17.9% (10.5) | 16.9% (10.2) | - 17.15% |
| Proportion of coverage of the target population by the PBF | 88.2% (16.2) | 86.7% (15.6) | 80.7% (17.1) | 89.1% (13.8) | 90.9% (12.8) | 91.1% (13.7) | 92.3% (13.0) | 92.0% (13.4) | 88.4% (15.6) | 89.3% (15.3) | 68.7% (34.7) | - 22.10% |
| Proportion of sanitation coverage | 67.7% (22.3) | 68.5% (22.0) | 69.2% (21.7) | 69.8% (21.5) | 70.5% (21.3) | 71.2% (21.2) | 72.0% (21.1) | 72.8% (21.0) | 73.6% (20.9) | 74.4% (20.9) | 75.2% (20.8) | 11.07% |
| Proportion of water coverage | 95.1% (5.7) | 94.6% (6.0) | 94.2% (6.5) | 93.8% (6.9) | 93.4% (7.4) | 93.1% (7.8) | 92.7% (8.2) | 92.4% (8.6) | 92.0% (8.9) | 91.7% (9.3) | 91.4% (9.7) | - 3.89% |
| Proportion of solid waste collection | 76.6% (17.2) | 77.7% (16.6) | 78.7% (16.1) | 79.7% (15.6) | 80.7% (15.2) | 82.0% (14.8) | 83.4% (14.5) | 84.8% (14.2) | 86.2% (13.9) | 87.5% (13.5) | 88.8% (13.1) | 15.92% |
| Proportion of coverage of the total population by the FHS | 64.1% (34.8) | 64.8% (33.4) | 69.2% (34.2) | 70.2% (34.1) | 71.8% (33.7) | 73.3% (33.3) | 74.0% (33.1) | 75.5% (31.6) | 77.7% (28.9) | 80.4% (27.0) | 80.4% (27.6) | 25.42% |
| Urbanization rate (%) | 73.7% (18.2) | 74.0% (18.0) | 74.2% (17.9) | 74.5% (17.7) | 74.7% (17.5) | 75.2% (17.5) | 75.7% (17.5) | 76.3% (17.4) | 76.9% (17.4) | 77.4% (17.4) | 78.0% (17.4) | 5.83% |
| Per capita monthly income in reais (R$)* | 471.4 (184.2) | 491.0 (192.9) | 485.4 (190.9) | 471.1 (182.4) | 622.3 (217.6) | 489.0 (188.8) | 483.5 (187.3) | 488.3 (113.9) | 491.7 (193.3) | 510.0 (202.3) | 492.8 (194.6) | 4.53% |
| Proportion of literate individuals | 76.5% (7.3) | 75.9% (7.3) | 74.5% (7.1) | 73.1% (7.6) | 71.3% (8.1) | 76.9% (6.4) | 75.3% (7.0) | 73.5% (7.5) | 71.7% (8.1) | 69.7% (8.7) | 67.5% (9.6) | - 11.76% |
| Data refer to the mean and (standard deviation). For income*, the median was considered. Causes of mortality in children under five years old are defined according to the International Classification of Diseases (ICD), 10th revision: diarrheal diseases (A00, A01, A02, A03, A04, A06-08) and malnutrition diseases (E40 - E46). Mortality rates are shown in the table for every ten thousand children up to five years old. N = number of municipalities. PBF=Bolsa Família Program. FHS=Family Health Strategy. | | | | | | | | | | | | |
|  |  |  |  |  |  |  |  |  |  |  |  |  |

Table S5: Descriptive measures of mortality rates by years of study and municipalities selected – Region South (N=777)

|  | **2006** | **2007** | **2008** | **2009** | **2010** | **2011** | **2012** | **2013** | **2014** | **2015** | **2016** | **Percentage Change 2006-2016** |
| --- | --- | --- | --- | --- | --- | --- | --- | --- | --- | --- | --- | --- |
|  |  |  |  |  |  |  |  |  |  |  |  |  |
| Mortality |  |  |  |  |  |  |  |  |  |  |  |  |
| Due to malnutrition | 0.31 (2.28) | 0.34 (3.49) | 0.30 (2.89) | 0.26 (3.23) | 0.25 (3.30) | 0.10 (1.28) | 0.03 (0.53) | 0.02 (0.23) | 0.09 (1.12) | 0.04 (0.78) | 0.10 (1.02) | - 67.74% |
| Due to diarrhea | 0.04 (0.64) | 0.07 (1.07) | 0.04 (0.73) | 0.07 (1.08) | 0.04 (0.51) | 0.09 (1.22) | 0.04 (0.59) | 0.02 (0.39) | 0.02 (0.35) | 0.04 (0.46) | 0.01 (1.79) | - 75.00% |
| Proportion of coverage of the total population by PBF | 17.1% (9.3) | 16.0% (9.2) | 13.8% (8.0) | 15.8% (9.4) | 15.7% (9.5) | 15.2% (9.5) | 15.4% (9.5) | 14.6% (9.2) | 13.7% (9.0) | 12.9% (8.6) | 11.5% (7.9) | - 32.74% |
| Proportion of coverage of the target population by the PBF | 90.4% (15.2) | 87.5% (16.7) | 82.3% (19.3) | 89.2% (16.8) | 89.2% (16.8) | 87.9% (18.0) | 89.6% (16.8) | 88.3% (18.0) | 84.3% (19.0) | 82.5% (20.2) | 63.7% (37.8) | - 29.53% |
| Proportion of sanitation coverage | 39.9% (27.2) | 40.9% (27.1) | 41.8% (27.3) | 42.8% (27.5) | 43.7% (27.8) | 45.0% (28.3) | 46.3% (26.8) | 47.7% (29.3) | 49.1% (29.8) | 50.6% (30.3) | 52.2% (30.9) | 30.82% |
| Proportion of water coverage | 93.0% (6.7) | 92.9% (6.3) | 92.8% (6.1) | 92.6% (6.3) | 92.5% (6.8) | 92.3% (7.3) | 92.0% (7.7) | 91.7% (8.1) | 91.3% (8.6) | 91.0% (9.0) | 90.7% (9.4) | - 2.47% |
| Proportion of solid waste collection | 68.0% (20.1) | 69.7% (19.5) | 71.4% (19.1) | 73.1% (18.7) | 74.8% (18.4) | 76.8% (18.3) | 78.9% (18.2) | 80.7% (18.0) | 82.5% (17.7) | 84.2% (17.4) | 85.6% (16.9) | 25.88% |
| Proportion of coverage of the total population by the FHS | 70.0% (34.0) | 72.2% (32.6) | 76.1% (32.1) | 76.4% (31.0) | 77.3% (30.4) | 78.7% (30.1) | 79.2% (29.7) | 80.5% (27.7) | 82.9% (24.2) | 86.3% (21.1) | 86.9% (20.8) | 24.14% |
| Urbanization rate (%) | 58.5% (23.4) | 59.0% (23.2) | 59.5% (23.0) | 60.0% (22.8) | 60.5% (22.7) | 61.2% (22.6) | 61.8% (22.5) | 62.5% (22.4) | 63.2% (22.4) | 63.9% (22.3) | 64.7% (22.3) | 10.59% |
| Per capita monthly income in reais (R$)* | 504.9 (167.9) | 517.5 (117.3) | 517.9 (176.7) | 502.2 (169.7) | 716.2 (215.8) | 525.6 (175.6) | 526.8 (174.3) | 543.0 (180.1) | 549.9 (181.1) | 573.4 (188.6) | 553.9 (182.0) | 9.70% |
| Proportion of literate individuals | 78.4% (7.6) | 77.8% (7.6) | 77.1% (6.9) | 75.6% (7.3) | 73.5% (7.9) | 78.2% (6.1) | 76.5% (6.5) | 74.6% (7.0) | 72.5% (7.6) | 70.3% (8.3) | 67.9% (9.0) | - 13.39% |
| Data refer to the mean and (standard deviation). For income*, the median was considered. Causes of mortality in children under five years old are defined according to the International Classification of Diseases (ICD), 10th revision: diarrheal diseases (A00, A01, A02, A03, A04, A06-08) and malnutrition diseases (E40 - E46). Mortality rates are shown in the table for every ten thousand children up to five years old. N = number of municipalities. PBF=Bolsa Família Program. FHS=Family Health Strategy. | | | | | | | | | | | | |
|  |  |  |  |  |  |  |  |  |  |  |  |  |
